# Endogenous Expression and Subcellular Localization of Core Apoptosis Regulators Reveal Key Differences Between Embryonic and Germline Apoptosis in C. elegans

**DOI:** 10.21203/rs.3.rs-8728396/v1

**Published:** 2026-02-05

**Authors:** Gokul Gopakumar, Afroza Aman, Stephane Rolland, Anton Gartner, Nadin Memar

**Affiliations:** Institute for Basic Science; Institute for Basic Science; UNIST; Institute for Basic Science; Institute for Basic Science

## Abstract

Apoptosis is a highly conserved form of programmed cell death controlled by a core molecular pathway that was first defined in *Caenorhabditis elegans* and is conserved in mammals. This pathway is composed of *egl-1/*BH3-only, *ced-9*/Bcl-2, *ced-4*/Apaf-1, and *ced-3/*Caspase. Despite being discovered more than 20 years ago, tissue-specific apoptosis induction as well as endogenous expression pattern and dynamic subcellular localization of apoptosis proteins remain incompletely defined. Here, we generated a complete set of CRISPR/Cas9-engineered transcriptional and translational reporters for all four apoptosis genes and systematically analyzed their expression and subcellular localization in the *C. elegans* germline and embryo.

We show that somatic apoptosis is driven by precise, lineage-specific activation of *egl-1*, whereas *ced-9*, *ced-4*, and *ced-3* are ubiquitously expressed. In contrast, DNA-damage triggers a robust CEP-1/p53-dependent-induction of *egl-1* throughout the germline, yet apoptosis occurs only in late pachytene cells. We also identify intron1 of *egl-1* as essential for CEP-1–dependent transcriptional activation. Analysis of *brc-1* and *syp-2* mutants demonstrates that distinct meiotic surveillance pathways converge on *egl-1* induction.

Analysis of the subcellular localization of the downstream regulators CED-9, CED-4, and CED-3 reveals dynamic, tissue-specific localizations that refine the classical apoptosis model. CED-4 transitions from a perinuclear distribution in the germline and early embryos to a predominantly mitochondrial localization later in embryogenesis, while CED-3 changes its subcellular localization depending on developmental stage and apoptotic status. CED-9 localizes to distinct mitochondrial foci in both embryo and germline.

Together, these reporters reveal that *C. elegans* apoptosis is governed by two mechanistically distinct programs: (1) lineage-specific *egl-1* activation in embryos and (2) checkpoint-mediated activation of *egl-1* in the germline, where additional, yet unidentified pathways restrict apoptotic execution. These reporters also provide a comprehensive toolbox for dissecting apoptotic and non-apoptotic functions of the conserved apoptotic machinery *in vivo*.

## Introduction

Apoptosis, the best-characterized form of programmed cell death, is a vital process that ensures proper development, maintains tissue homeostasis, and eliminates damaged cells in multicellular organisms (1–3). Genetic studies in *Caenorhabditis elegans* identified the evolutionarily conserved core components of the apoptotic pathway, which were later found to be conserved in mammalian cells (4). While *egl-1* encodes a BH3-only protein that functions as the most upstream pro-apoptotic factor, *ced-9* encodes a Bcl-2–like anti-apoptotic protein. *ced-4* encodes an Apaf-1–related pro-apoptotic protein lacking the cytochrome c–binding domain, and *ced-3* encodes a caspase related to both initiator caspase-9 and executioner caspase-3 (4–11). While the apoptotic machinery is conserved in mammals, how apoptosis is spatially and temporally regulated during development remains incompletely understood (1). The genetic tractability and essentially invariant cell lineage of *C. elegans* make it an ideal system to dissect these mechanisms.

During *C. elegans* development, 131 of the 1090 somatic cells are programmed to die (12, 13), raising the question of how these 131 cells, which arise from multiple cell lineages, are selectively destined to die while their sisters survive. Cell-specific apoptosis induction largely depends on the transcriptional upregulation of *egl-1*, which is controlled by *cis*-regulatory sequences located upstream and downstream of its transcription start site (14). Several transcription factors have been shown to regulate *egl-1* expression in a cell-specific manner (5, 9, 11, 14, 15). In contrast, *ced-9*, *ced-4*, and *ced-3* are expressed broadly, including in cells that do not undergo apoptosis (16–22). Post-transcriptional mechanisms have also been shown to fine-tune the expression of *egl-1* (ref. 23, 24), as well as *ced-9* and *ced-3* (ref. 25).

The lack of suitable antibodies and endogenous reporters has hampered the systematic analysis of the expression of core apoptosis machinery genes during embryogenesis, and this is especially the case for *egl-1*. Recent work has begun to overcome these limitations: Lambie and colleagues (16) and Tucker and colleagues (26) used CRISPR/Cas9–mediated tagging of endogenous *ced-9*, *ced-4*, and *ced-3* loci to visualize their expression and subcellular localization in live embryos. In contrast, comparable endogenous reporters for *egl-1* and systematic analyses of its embryonic expression is still lacking.

The adult germline is the only proliferative tissue in *C. elegans*, and apoptosis is restricted to female germ cells during late meiotic pachytene. A baseline level of physiological germ-cell apoptosis removes approximately half of the germ cells in an *egl-1*–independent manner to maintain tissue homeostasis (27). By contrast, germ-cell apoptosis triggered by DNA damage or defects in meiotic recombination (28) are dependent on *egl-1* and its transcriptional induction by the *C. elegans* CEP-1/p53-like transcription factor (29–32). Immunostaining revealed broad cytoplasmic localization of EGL-1::V5 translational fusion protein in the late pachytene region upon ionizing radiation IR (33), whereas CED-9 (ref. 17), CED-4 (ref. 17), and CED-3 (ref. 19, 22) are constitutively expressed. However, expression of these genes in the germline has not been systematically characterized, and it remains unclear whether the localization of these proteins dynamically changes upon induction of apoptosis or during specific apoptotic stages. Notably, reliable transgene germline expression requires single-copy integration at endogenous loci to minimize silencing (34).

According to the classic model of apoptosis, CED-9 inhibits apoptosis in healthy cells by binding a CED-4 dimer at the outer mitochondrial membrane (OMM) (35). Upon apoptotic induction, EGL-1 binds to CED-9, inducing a conformational change of CED-9, triggering the release of CED-4, which then assembles into the apoptosome at the perinuclear membrane to activate the CED-3 caspase (36, 37). However, several observations challenge this model and suggest that it requires refinement. CED-4 and CED-3 remain localized at mitochondria during mid-embryogenesis in both healthy and apoptotic cells (16). Although perinuclear CED-4 localization has been reported in *ced-9* loss-of-function (lf) mutants, and upon *egl-1* overexpression (17, 26, 38), this redistribution is not consistently observed (16). Additionally, CED-4 exhibits a perinuclear localization in all germ cells, including those not undergoing apoptosis (17, 20, 21, 38).

Here, we describe the developmental expression patterns and subcellular localizations of *C. elegans* apoptosis genes, *egl-1, ced-9, ced-4* and *ced-3*, using transcriptional and translational reporters generated by CRISPR/Cas9 at their endogenous loci. A central unresolved question is how transcriptional activation of apoptosis genes is translated into protein abundance, localization, and apoptotic execution. Using these reporters, we dissect how transcriptional output, protein localization, and tissue-specific competence together shape apoptotic outcome in the germline and embryos.

## Materials and Methods

### General C. elegans Maintenance and Strains

*C. elegans* strains were cultured and maintained at 15°C and 20°C unless stated otherwise (39). The Bristol N2 strain was used as the wild-type strain. Worms were kept on nematode growth medium (NGM) plates seeded with OP50 bacteria (~ 100 μl per plate). The strains used in this study are listed in Table S2. All translational reporter strains were maintained as homozygotes. All transcriptional reporter strains are homozygous viable and behave as null alleles. The *ced-9(syb5190)* transcriptional reporter is viable only in the *ced-3(n717)* mutant background.

#### CRISPR/Cas9 Genome Editing

As indicated in Table S2, some genome edits were generated by Sunybiotech. *egl-1(gt3323), egl-1(gt3399), egl-1(gt3361), ced-9(gt3374), ced-4(gt3372), syp-2(gt3637)* and *brc-1(gt3334)* were generated in the Gartner laboratory using CRISPR/Cas9 gene editing following established methods (40). For the generation of each genome edit, ~10 young adult hermaphrodites were injected into one or both gonad arms. They were then recovered individually into 5 μl of M9 buffer in the center of the OP50-seeded plate. Recovered worms were then maintained at 15°C. F1 progenies that displayed the roller phenotype were singled 5–7 days post-injection and subsequently screened by pheno- and genotyping, using polymerase chain reaction (PCR) and/or sequencing after being permitted to lay eggs for 24–48 hours. For the edits generated in the Gartner laboratory, the sequences of the CRISPR RNA (crRNA), single-stranded OligoDeoxyNucleotide (ssODN), and primers for the amplification of double stranded DNA (dsDNA) fragments are shown in Table S3.

#### Generation of Transcriptional and Translational Reporters

Fluorescent protein sequences (eGFP and mKate2) were obtained from the Frøkjær-Jensen laboratory, and a codon-optimized tdTomato sequence was used as previously described (41, 42). To ensure germline expression and minimize silencing, a synthetic Periodic An/Tn Cluster (PATC) intron was inserted into each reporter (Table S1, Fig. S1-S3) ref. (34). For the transcriptional reporters, an SV40 nuclear localization sequence (NLS) was placed at the N-terminus and an *egl-13* NLS at the C-terminus, separated from the fluorescent protein coding sequence by linker sequences (Table S1, Fig. S1-S3). To enable efficient exchange of fluorescent markers, guide RNA target sites were introduced flanking the fluorescent protein coding region (Fig. S1-S3, Table S3).

### Ionizing Radiation (IR) Treatment and Preparation of of C. elegans Germlines for Live Imaging

A synchronized population of L4-staged worms was obtained by performing a layoff. In short, 15–20 adult hermaphrodite worms were transferred to a medium plate and allowed to lay eggs for 6 hours at 20°C. The adult worms were removed, and the plates were then incubated at 20°C or 15°C until the worms reached the L4 stage. Synchronized L4-staged worms were then treated with a 0 Gy or 90 Gy dose of IR using a Biological X-ray irradiator (Rad Source; RS-2000). 24 hours post-IR treatment, the adult hermaphrodites were transferred onto a 2% agar pad into 10 ul of 5 mM tetramisole to immobilize the worms. An 18 × 18 mm coverslip (1.5H Zeiss) was placed on top and sealed with (for confocal and longterm live imaging) or without (for 4D microscopy germline imaging) vaseline to prevent desiccation. Live imaging of the *C. elegans* germline was performed as described below.

### Confocal Image Acquisition of C. elegans Germlines

Z-stacks of the entire germline were acquired using a laser scanning confocal microscope (LSM880, Carl Zeiss) with either a 40x/1.2 NA water-immersion objective or a 63x/1.4 NA oil-immersion objective, and the Zen 2.3 SP1 software (Zeiss). The imaging settings are listed in Table S4. Image analysis was performed using Fiji software, and the following LUT settings were used (Table S5). ‘n’ represents the number of germlines analyzed.

### Spinning Disk Confocal Long-term Live Imaging of C. elegans Germlines

Live imaging of the *zhIs198 [Plim-7::Δpes-10::mCherry::PH(PLC1delta1)::unc-54 3’UTR] I; egl-1(gt3361) V* (43) animals was performed using a spinning disk confocal microscope (ECLIPSE Ti2-E, Nikon) with spinning disk head (CSU-W1, Yokogawa Electric Corporation) and the NIS element software (Nikon). A 60x/1.4 NA oil objective was used to capture the images. The acquisition parameters used were 488 nm laser at 40% intensity, 561 nm laser at 25% intensity, 300 ms exposure time, no binning, and image capture every 5 minutes for a total duration of 2 hours. Image and video analysis were carried out in Fiji using the following LUT settings: Magenta (mCherry) – (105–150); Green (eGFP) – (105–150).

### 4D microscopy Image Acquisition of C. elegans Germlines

The Z-stack of the entire germline was captured using a 4D microscope (Axio Imager M2, Zeiss) and Time to Live software (Caenotec) (44, 45). A 100x/1.3 NA oil objective was used to capture the images. For eGFP the following acquisition parameters were used: LED intensity: 15%; exposure time: 150 ms; binning: ON. Image analysis was performed using Fiji software, and the min and max (brightness) were adjusted to 0–255 for all the images, except when indicated otherwise in the figure legends.

### C. elegans Germline Apoptosis Counting

A synchronized population of L4-staged worms was obtained by filtering L1 worms from freshly starved plates, as previously described (46). In short, from a freshly starved medium NGM plate, worms were washed using 2 ml of M9. The solution was then collected in a 20 ml syringe and passed through a nylon mesh (11 μm Nylon net filters, Millipore), with holes large enough only to allow L1 larvae to pass. The L1 worms were then transferred to an NGM medium plate with OP50 bacteria using a glass pipette and allowed to dry. The plates were then incubated at 20°C or 15°C until the worms reached the L4 stage. Apoptotic cell corpses in the germline were quantified using Nomarski optics as described previously (27). Briefly, the synchronized population of L4-staged worms was exposed to 0 Gy or 90 Gy dose of IR (using a Biological X-ray irradiator (Rad Source; RS-2000)). 24-, 36-, and 48-hours post-IR treatment, the adult hermaphrodites were transferred onto a 2% agar pad containing a drop of 5 mM tetramisole to immobilize the worms. An 18 × 18 mm coverslip (1.5H Zeiss) was placed on top, and the number of cell corpses per gonad arm was scored using a 4D microscope/Axio Imager M2 microscope (Zeiss) using a 100x/1.3 NA oil objective. All quantifications were performed blind.

### Experiment to Monitor Time and Dose Dependency of the egl-1 Reporter Induction by IR

For time-course analysis of the *egl-1(gt3323)* and *egl-1(gt3361)* animals, L4-staged worms synchronized by filtration were irradiated with 90 Gy dose of IR, and imaged using a 4D microscope/Axio Imager M2 microscope (Zeiss) equipped with a 100x/1.3 NA oil objective at specified time points post-irradiation (0-, 0.5-, 1-, 2-, 3-, 4-, 8-, 12-, and 24-hours). For eGFP the following acquisition parameters were used: LED intensity: 15%; exposure time: 150 ms; binning: ON. Image analysis was performed using the Fiji software, and the min and max (brightness) were adjusted to 0–255 for all the images.

For dose-response analysis, L4-staged worms were treated with 0 Gy, 30 Gy, 60 Gy, or 90 Gy, and imaged 24 hours post-IR treatment using a 4D microscope/Axio Imager M2 microscope (Zeiss) equipped with a 100x/1.3 NA oil objective. For eGFP the following acquisition parameters were used: LED intensity: 15%; exposure time: 150 ms; binning: ON. Image analysis was performed using the Fiji software, and the min and max (brightness) were adjusted to 0–255 for all the images.

### Immunostaining of the C. elegans Germline

Immunostaining of the *C. elegans* germline was performed as previously described (47). Briefly, adult hermaphrodites (24 hours post-L4 stage) were transferred to a glass dish containing phosphate-buffered saline (PBS) + 0.2 mM tetramisole. The heads of the worms were cut off using a blade, and the gonads were collected into a 1.5 ml low protein-binding Eppendorf tube using a Pasteur pipette. Immediately after transfer, 1 ml of 3.7% formaldehyde in PBS was added, and samples were incubated at room temperature for 10 minutes. Following fixation, the gonads were washed with PBST (PBS containing 0.1% Tween-20) and post-fixed in 1 ml of 100% methanol for 5 min. After three quick washes with PBST, the gonads were permeabilized in 500 μl of PBS containing 1% Triton X-100 for 10 minutes at room temperature; this step was repeated four times. Samples were then washed twice with PBS and incubated overnight (~12–14 hours) in blocking buffer PBSTB (PBST + 1 mg/ml bovine serum albumin (BSA)). Primary antibodies [anti-Mitomix (mouse anti-ATP5A, anti-Cytochrome C, and anti-PDHA1; 1:200; Abcam) (48) and rabbit anti-HA (1:1000; Cell Signaling Technology)] in PBSTB were added, and samples were incubated for approximately 12 hours at 4°C. Gonads were subsequently washed five times with PBST for 5 minutes each and incubated overnight at 4°C with secondary antibodies [Alexa Fluor 488-conjugated goat anti-rabbit (1:5000; Invitrogen) and Alexa Fluor 594-conjugated donkey anti-mouse (1:500; Invitrogen)] in PBSTB. After five additional washes with PBST (5 minutes each), 20 μl of Vectashield with DAPI (Vector Labs) was added to the tube containing the gonads. Gonads were then transferred onto a large 2% agarose pad, covered with a 22 × 22 mm coverslip, and incubated at 4°C overnight to allow drying. Slides were finally sealed with nail polish.

For image acquisition, the gonads were analyzed using a high-resolution confocal laser scanning microscope (LSM 980; Carl Zeiss). A 40x/1.2 NA water objective was used to capture the images. The images were acquired using the following settings: 488 nm laser intensity: 1%; Master Gain: 800 V; Digital Gain: 1.0; 561 nm laser intensity: 1%; Master Gain: 690 V; Digital Gain: 1.0; 405 nm laser intensity: 1%; Master Gain: 750 V; Digital Gain: 1.0.

### 4D Microscopy of the C. elegans Embryo

The method for 4D microscopy was described previously (45). Modifications of this system are described in (44). All recordings were acquired at 25°C.

#### Lineage Analysis

Lineage analysis of the *C. elegans* embryo was performed as previously described (44, 45). All 4D-recordings generated were analyzed using the Software Database SIMI©BioCell (SIMI Reality Motion Systems, Unterschleissheim, Germany; http://www.simi.com/). The observer follows cells and the coordinates are recorded approximately every 2 minutes. The cell cleavages are assessed by marking the mother cell before the cleavage furrows ingress and subsequently by marking the centers of the daughter cells three frames later (105 seconds). By marking every cell throughout embryonic development, the complete cell lineage of an embryo is generated. These data can be used to create 3D representations of all nuclear positions at any given developmental stage.

For the analysis of apoptotic events, we tracked the first 13 apoptotic events from the AB lineage and the MSpaapp apoptotic event. GFP scans are indicated by horizontal green lines in Fig. 3.

### Immunostaining of the C. elegans Embryo

Immunostaining of the *C. elegans* embryo was performed as previously described (49). Microscope slides were cleaned with 70% ethanol. Following this, each microscope slide was heated on a hot plate at 80°C and then coated twice with poly-L-lysine (0.25 mg/ml), with drying in-between at 80°C. After the second poly-L-lysine coating, the slides were cooled down on the bench before the next step. *C. elegans* embryos were then transferred using a P10 pipette onto the coated microscope slide. A new 18 × 18 mm coverslip (1.5H Zeiss) was then placed on top of the area where the embryos were transferred (at ~45° angle, with the corner of the coverslip hanging over the edge of the coated microscope slide). The microscope slide was then immediately flash-frozen on dry ice (for at least 30 minutes). The coverslip was then very quickly removed by flicking it (freeze–crack method), and the slides were incubated in 100% methanol for 5 minutes, followed by 100% acetone for 5 minutes. Following the fixation, the slides were air-dried (for ~8 minutes) before being stored at − 20°C if the next step was not performed right away. Permeabilization of the embryos was performed by incubating the slides 4 times for 10 minutes in PBS containing 1% Triton X-100 at room temperature. Slides were then washed twice with PBS and incubated overnight at 4°C (~12–14 hours) in blocking buffer PBSTB.

Primary antibody [anti-GFP rabbit (Abcam Ab290) 1:500, anti-Mitomix (mouse anti-ATP5A, anti-Cytochrome C, and anti-PDHA1; 1:200; Abcam) (48) and rabbit anti-HA (1:1000; Cell Signaling Technology)] (49) in PBSTB was added to the embryos, and incubation was performed overnight at 4°C in a humid chamber. After three washes with PBST for 10 minutes, a secondary antibody Alexa Fluor 594-conjugated donkey anti-mouse (1:500; Invitrogen), Alexa Fluor 488-conjugated donkey anti-rabbit (1:500; Invitrogen) in PBSTB was added to the embryos, and incubation was performed overnight at 4°C in a humid chamber. After two washes with PBST and one wash with PBS, post-fixation with PBS + 3.7% PFA (Paraformaldehyde) was performed for 10 minutes. The slide was then washed with PBS for 10 min, with PBST (twice for 10 minutes each), and with PBS for 10 min. Afterwards, except for the area containing the embryos, the rest of the slide was dried using a KIMTECH tissue. 10 μl of Vectashield with DAPI (Vector Labs) was then added to the area containing the embryos, and an 18 × 18 mm coverslip (1.5H Zeiss) was placed on top. Nail polish (clear) was then used to seal the slides, with the nail polish allowed to dry in the dark. Once all slides were sealed correctly, they were then placed at 4°C until analysis.

For image acquisition, embryos were analyzed using a high-resolution confocal laser scanning microscope (LSM 980; Carl Zeiss). A 63× oil objective (1.4 NA) was used to capture the images, with a Z-stack of 0,13 μm per step used. Embryos were sequentially illuminated by a 465 nm laser (for DAPI), a 488 nm Laser (for Alexa 488 anti-rabbit), and a 561 nm Laser (for Alexa 594 anti-mouse).

#### Analysis of Embryonic Lethality and Brood Size

Analysis of brood size and embryonic lethality was performed as previously described (50). Briefly, L4 worms were picked and either maintained at 15°C and 25°C. The worms were transferred to fresh small (35 mm) plates with food twice a day (morning and evening) until they no longer laid eggs. Shortly after transferring the worms to a fresh plate, the number of eggs laid was counted. After 24–36 hours, the number of dead eggs was counted and after 24–48 hours, the number of animals hatched was counted.

## Results

We generated transcriptional and translational reporters at the endogenous loci using CRISPR/Cas9 to measure physiological steady-state gene expression and protein subcellular localization (as described in Materials and Methods) (Table S1 + S2, Fig. S1-S3).

### IR-Induced egl-1 Expression in the Germline Requires the First Intron of egl-1

**Figure 1:**
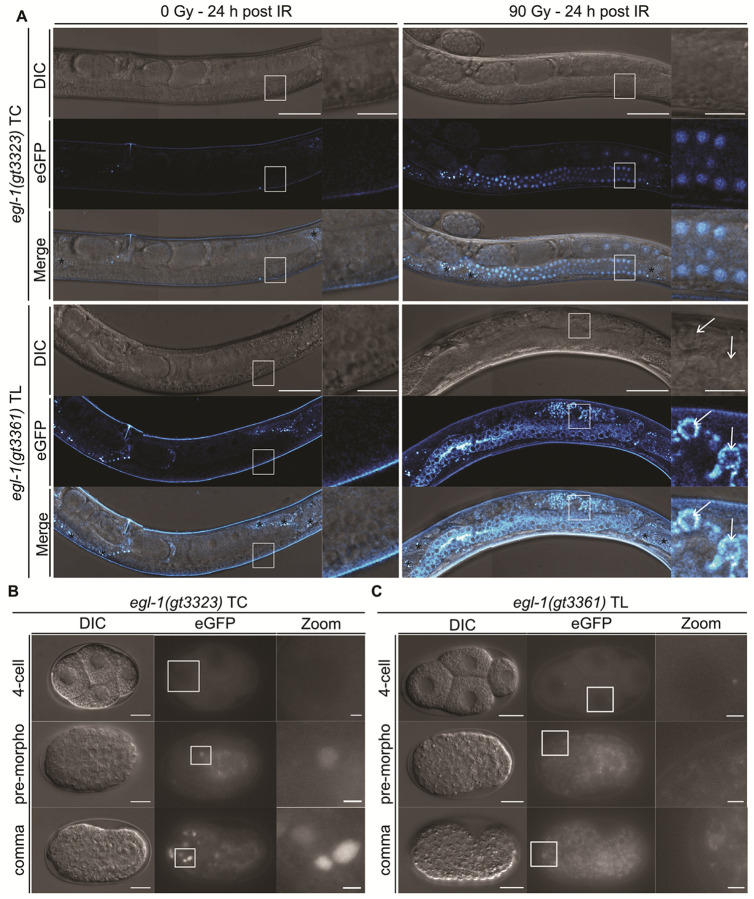
Expression pattern of the transcriptional *egl-1(gt3323)*TC and translational *egl-1(gt3361)* TL reporters in the germline and embryo. **(A)** Confocal live imaging of the transcriptional *egl-1(gt3323)* TC and translational *egl-1(gt3361)* TL reporters in the germline with and without IR. L4-staged worms were exposed to 0 Gy or 90 Gy of IR and imaged 24 hours post-treatment. Insets show zoomed regions outlined by white boxes in the main panels. Asterisks (*) mark intestinal autofluorescence. White arrows indicate apoptotic germ-cell corpses. Scale bars: 50 μm (main panels), 10 μm (insets). Expression of the **(B)** transcriptional *egl-1(gt3323)* TC reporter and the **(C)** translational *egl-1(gt3361)*TL reporter during embryogenesis (4-cell, pre-morphogenesis, comma stage). Insets show zoomed regions outlined by white boxes in the main panels. Scale bars: 10 μm (main panels), 2 μm (insets).

To investigate *egl-1* transcription, we designed two transcriptional reporters. The *egl-1(syb4530)*, replaces the entire open reading frame with our NLS::linker::eGFP::linker::NLS cassette, whereas *egl-1(gt3323)*, retains exon1, intron1, and the first codon of exon2 (Fig. S4A). Although *egl-1* transcription is induced by IR (51), only *egl-1(gt3323)* supported IR-induced expression in the germline (Fig. 1A and S4C/C’). We further show that this induction is time- and dose-dependent (Fig. S5 + S6). Loss of reporter expression in a *cep-1(lg12501)*-deficient background confirmed that IR-induced *egl-1* transcription is CEP-1-dependent (Fig. S4C/C’) (51). Consistent with the presence of canonical p53-consensus binding sites within intron1 (Fig. S4A + B) (52), IR-induced expression was abolished in *egl-1(gt3399)*, which lacks intron1 (Fig. S4A+S4C/C’). Together, these data identify intron1 as a CEP-1–responsive regulatory module required for IR-induced *egl-1* transcription.

### IR-Induced egl-1 Expression in the Germline is Ubiquitous, but Apoptosis Only Occurs in a Subset of Cells

**Figure 2:**
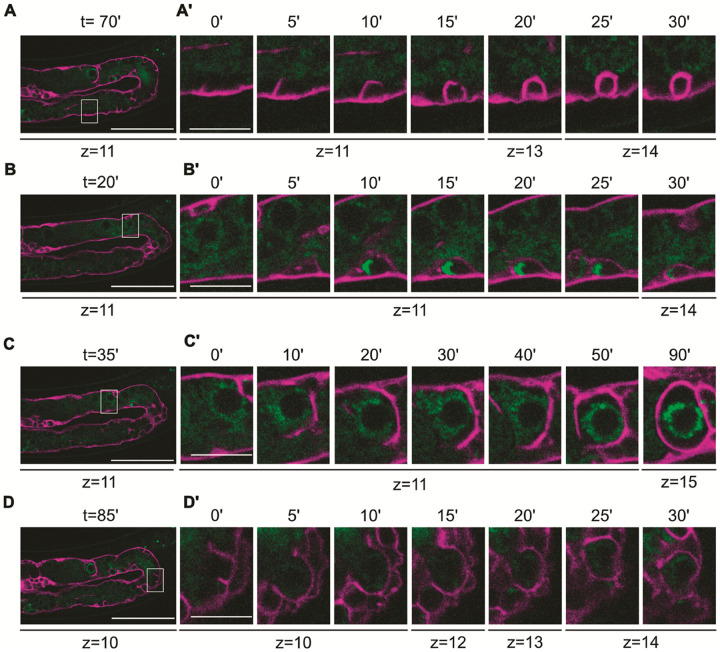
Hyperaccumulation of EGL-1 in a subset of cell corpses. **(A-D)** Snapshots from long-term spinning-disk confocal imaging of germlines from *zhls198* [Plim-7::Δpes-10:: mCherry::PH(PLC1delta1)::unc-54 3’UTR]*; egl-1(gt3361)* animals, imaged 24 hours after exposure to 90 Gy of IR. EGL-1 is visualized in green, and the membrane-localized engulfment marker in magenta. ‘z’ indicates the focal plane of the z-stack shown, and ‘t’ indicates the elapsed timepoint from the start of the recording in minutes. Insets showed zoom regions outlined by white boxes in the main panels. **(A’-D’)** Time-lapse series for different corpses. The first image was set to t=0 to highlight the engulfment duration. **(A’)**Small apoptotic corpse that lacks EGL-1 entirely, consistent with physiological germ-cell apoptosis. **(B’)** Small apoptotic corpse that shows an increase in EGL-1, followed by a decrease. **(C’)** Large apoptotic corpse that exhibits a marked and sustained increase that culminates in EGL-1 hyperaccumulation. **(D’)**Large apoptotic corpse with moderate EGL-1 expression, which does not reach hyperaccumulated levels as in **C’**.

IR-induced germ-cell apoptosis is blocked in the *egl-1(gt3323)* transcriptional reporter, whereas *egl-1-*independent physiological germ-cell apoptosis remains unperturbed (Fig. S8A) (27, 48). Wild-type levels of IR-induced germ-cell apoptosis in the *egl-1(gt3361)* translational reporter demonstrates the reporter’s functionality (Fig. S8A). We further show this reporter is induced by IR in a time- and dose-dependent manner (Fig. S5 + S7). Confocal imaging revealed that EGL-1 is ubiquitously and uniformly expressed upon IR throughout the mitotic, and meiotic part of the germline up to late pachytene, with heterogeneous expression emerging in late pachytene and early diakinesis (Fig. 1A). Long-term spinning-disk confocal imaging (~ 2 hours (n = 3)) using the CED-1 engulfment reporter (*zhIs198*) to mark apoptotic corpses uncovered diverse EGL-1 protein dynamics among apoptotic germ cells (43). Small apoptotic corpses lacking EGL-1 entirely, consistent with *egl-1*-independent physiological germ-cell apoptosis (Fig. 2A/A’), (27, 48). Small apoptotic corpses showing an increase in EGL-1, followed by a decrease, EGL-1 appearing to congregate on one side of the corpse (Fig. 2B/B’). Large apoptotic corpses exhibiting a marked and sustained increase of EGL-1 leading to hyperaccumulation (Fig. 2C/C’). Large apoptotic corpses with moderate EGL-1 expression, not showing hyperaccumulation (Fig. 2D/D’, Movie S1-S4). Thus, our observations reveal that EGL-1 protein dynamics varies substantially between apoptotic germ cells. Notably, EGL-1 is also expressed in non-apoptotic cells. In summary, our findings show that IR robustly induces *egl-1* in a CEP-1-dependent manner throughout the germline and that this induction is necessary (51, 53), but not sufficient for IR-induced apoptosis, which occurs only on late pachytene cells. Thus, mechanisms unrelated to EGL-1 induction specify which cells undergo apoptosis.

### Recombination and Chromosome Pairing Defects Induce egl-1 Expression

Defects in meiotic recombination and chromosome pairing induce germ-cell apoptosis (54–57). We, therefore, examined *egl-1* induction in *brc-1*/BRCA1 and *syp-2* mutants, which are defective in recombinational repair and meiotic chromosome pairing, respectively (58–60). *egl-1* was induced in both mutants, even in the absence of IR. In *brc-1* mutants, *egl-1* expression was detected in both mitotic and meiotic regions of the germline, consistent with activation of the DNA damage checkpoint (Fig. S9A). In contrast, *egl-1* expression in *syp-2* mutants was restricted to the pachytene region (Fig. S9B), consistent with defects in chromosome synapsis that delay repair of meiotic double-strand breaks via inter-sister recombination (58, 59). Thus, disruptions in either DNA repair or chromosome pairing activate distinct checkpoint signals that converge on *egl-1* induction to eliminate defective germ cells.

### Embryonic Lineage Analysis Reveals Precise Coupling between egl-1 Expression and Apoptosis

**Figure 3:**
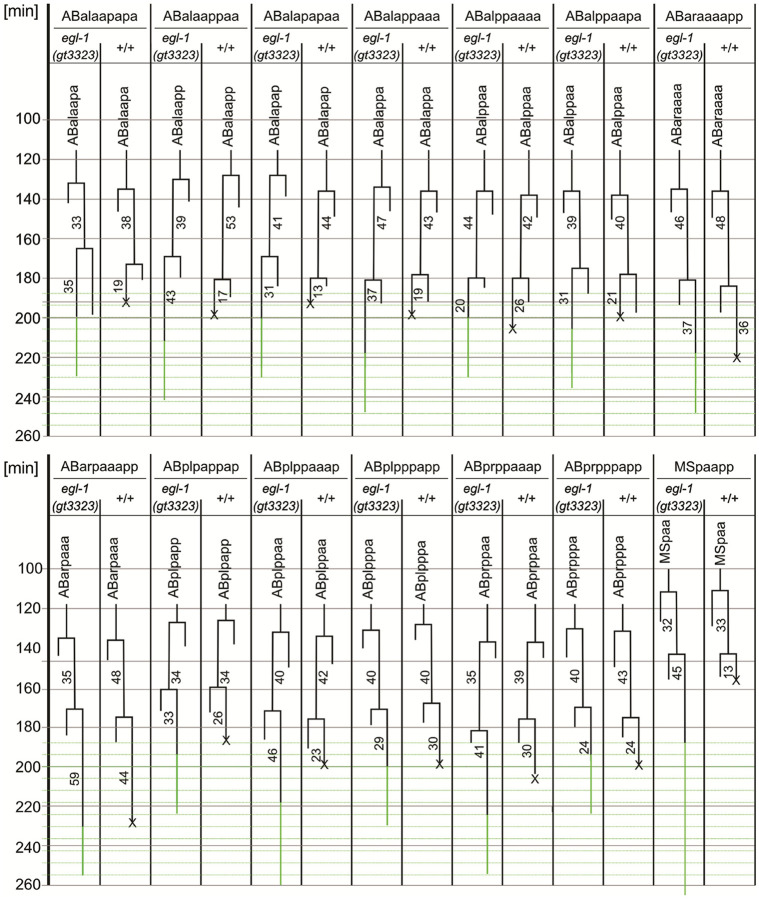
Lineage analysis of the transcriptional *egl-1(gt3323*) reporter strain and wild type (+/+). Embryos of both the transcriptional *egl-1(gt3323)* reporter strain and wild type (+/+) were recorded side-by-side, and eGFP scans were started after 188 minutes and taken every 6 minutes afterwards (as indicated by the horizontal green lines). Lineaging was performed on the first 13 AB-derived cell deaths, starting after the 9th round of cell division, and on the MSpaapp cell death. The time point at which a cell dies in the wild type is indicated by a cross (X). For the transcriptional *egl-1(gt3323*) reporter the time point at which eGFP expression is detectable, is marked by a vertical green line. The cell cycle length of mother cells of each future cell destined to die is indicated. In addition, the time between the birth of a cell until its cell corpse formation (in wild type) or until eGFP expression starts (in the *egl-1(gt3323*) reporter strain) is indicated.

To test whether *egl-1* expression correlates with apoptosis induction during embryogenesis, we performed cell lineage analysis of the first 13 apoptotic deaths occuring after the 9th round of cell division in the AB lineage and the MSpaapp death. The transcriptional *egl-1(gt3323)* reporter was specifically expressed in cells programmed to die, but apoptosis is blocked since *egl-1(gt3323)* is a null allele (Fig. 1B + 3, S10A). Comparing wild-type embryos and those carrying the transcriptional reporter side-by-side revealed that reporter expression generally initiated after apoptotic corpse formation (Fig. 3). For example, the ABalapapaa corpse forms 13 minutes after birth of the cell in wild-type embryos. Reporter expression begins ~ 31 min after birth in the transcriptional *egl-1(gt3323)* reporter, likely reflecting low initial *egl-1* expression levels and/or eGFP maturation time. The translational *egl-1(gt3361)* reporter was detected in cells programmed to die without blocking apoptosis, indicating full functionality (Fig. 1C+S10A). Altogether, these findings show that cell-specific *egl-1* activation closely matches the execution of apoptosis.

#### EGL-1 Mitochondrial Localization in the Germline and During Embryonic Apoptosis

**Figure 4:**
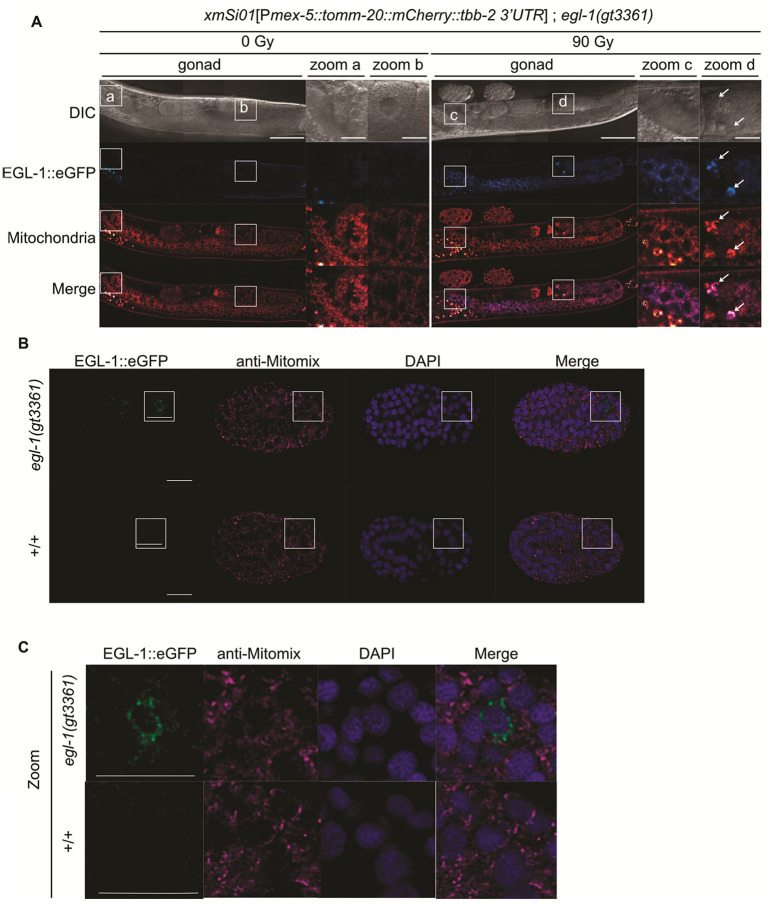
Localization of the EGL-1 protein in the germline and embryo. **(A)** Confocal live imaging of the translational *egl-1(gt3361)*TL reporter with the mitochondrial reporter (*xmSi01*[P*mex-5::tomm-20::mCherry::tbb-2 3’UTR*]) in the germline with and without IR. L4-staged worms were exposed to 0 Gy or 90 Gy of IR and imaged 24 hours post-treatment. Insets show zoomed regions outlined by white boxes in the main panels. White arrows indicate apoptotic germ-cell corpses. Scale bars: 50 μm (main panels), 10 μm (insets). **(B)** Airyscan imaging of immuno-stained *egl-1(gt3361)* translational TL reporter embryos at the pre-morphogenetic stage. Embryos were stained with anti-GFP and anti-Mitomix antibodies. Insets show zoomed regions outlined by white boxes in the main panels. Scale bars: 10 μm (main panels). **(C)** Zoom of inset shown in (**B)**. 10 μm (insets).

We analyzed the subcellular localization of the EGL-1 protein in the germline and during embryogenesis. Upon IR, EGL-1 colocalized with mitochondria throughout the entire germline (Fig. 4A+ S13). A subset of apoptotic corpses, identified by button-like morphology under DIC optics, exhibited pronounced EGL-1 hyperaccumulation. However, its functional significance remains unclear (Fig. 4A). Because these corpses were already undergoing engulfment, and mitochondria in conjunction with EGL-1 congregated on one side of the corpse, they likely represent corpses during late-stage of engulfment (Fig. 2B’).

In embryos, high-resolution confocal microscopy of fixed samples revealed single cells with intense EGL-1 staining that colocalizes with mitochondria (Fig. 4B + C). We hypothesize that these cells are cells destined to die.

### Ubiquitous ced-3 Expression with Apoptosis-Specific Changes in CED-3 Localization

To systematically study *ced-3* expression, we generated a transcriptional reporter, *ced-3(syb5182)*, replacing the entire coding sequence, and a translational reporter, *ced-3(syb5180)*, with a C-terminal linker::eGFP sequence to tag both CED-3 isoforms (Fig. S1B). As expected, germline and embryonic apoptosis were abolished in the transcriptional *ced-3(syb5182)* reporter, phenocopying a strong *ced-3(*lf*)* mutant. In contrast, apoptosis occurred at wild-type levels in the translational *ced-3(syb5180)* reporter, confirming full functionality (Fig. S8D+S10A).

**Figure 5:**
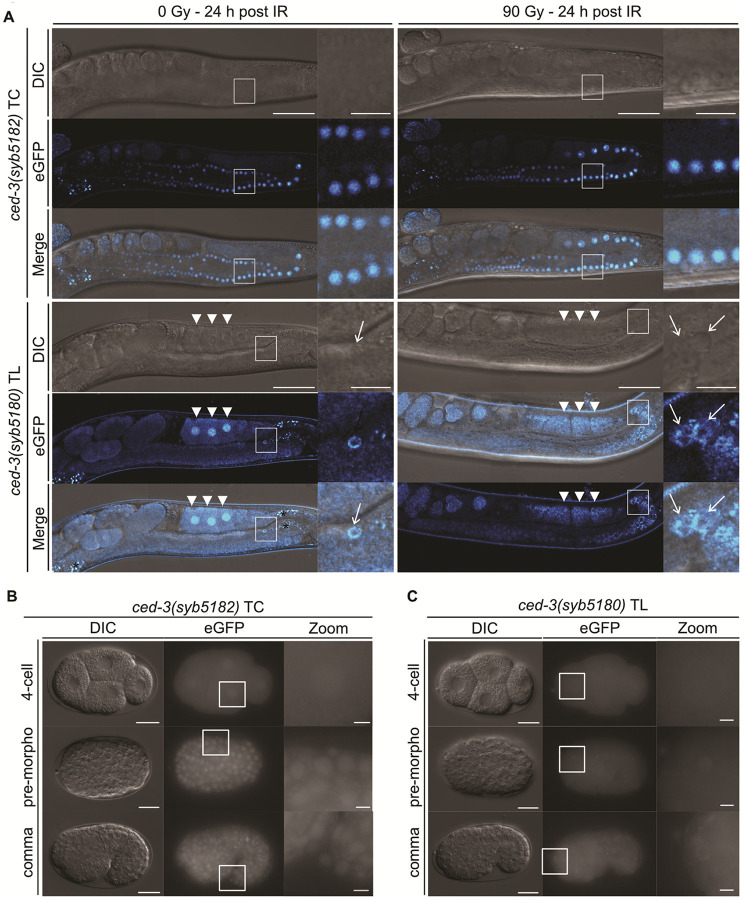
Expression pattern of the transcriptional *ced-3(syb5182)*TC and translational *ced-3(syb5180)* TL reporters in the germline and embryo. **(A)** Confocal live imaging of the transcriptional *ced-3(syb5182)* TC and translational *ced-3(syb5180)* TL reporters in the germline with and without IR. L4-staged worms were exposed to 0 Gy or 90 Gy and imaged 24 hours post-treatment. Insets show zoomed regions outlined by white boxes in the main panels. Asterisks (*) mark intestinal autofluorescence. White arrows indicate apoptotic germ-cell corpses. White arrowheads indicate oocytes. Scale bars: 50 μm (main panels), 10 μm (insets). Expression of the **(B)**transcriptional *ced-3(syb5182)* TC and **(C)** translational *ced-3(syb5180)* TL reporters during embryogenesis (4-cell, pre-morphogenesis, comma stage). Insets show zoomed regions outlined by white boxes in the main panels. Scale bars: 10 μm (main panels), 2 μm (insets).

Both reporters are ubiquitously expressed with and without IR in the germline (Fig. 5A). Without IR, the CED-3 protein displays a diffuse localization in both the cytoplasm and the nucleus (Fig. 5A). Upon IR, CED-3 localization remained unchanged in 4/9 animals (Fig. S11B), but redistributed to structures around the nuclear periphery and in the cytoplasm in 5/9 animals. These structures form a pattern broadly similar to the endoplasmic reticulum (ER) (61). Notably, CED-3 also accumulated in the cytoplasm of some late-stage apoptotic corpses in both irradiated and unirradiated germlines (Fig. 5A, arrows).

In unirradiated germlines, CED-3 localizes to the nuclei of diplotene-stage oocytes, with increasing nuclear enrichment in late-stage oocytes (Fig. 5A, arrowheads). Colocalization with the H2B-reporter (*ltls37)* confirmed association of CED-3 with condensed meiotic chromosomes in proximal oocytes (Fig. S11A).

During embryogenesis, the transcriptional *ced-3(syb5182)* and translational *ced-3(syb5180)* reporters are ubiquitously expressed (Fig. 5B + C) consistent with previous reports (16). Interestingly, the translational *ced-3(syb5180)* reporter localizes to both the cytoplasm and the nucleus in all cells. In apoptotic corpses, it adopts a cytoplasmic ring-like pattern, indicating a shift toward predominantly cytoplasmic localization (Fig. 5C).

In summary, CED-3 is expressed ubiquitously in the germline and embryos and its localization changes upon induction of apoptosis.

CED-4 Subcellular Localization Differs Between the Germline and Embryo and Changes Dynamically During Embryogenesis.

**Figure 6:**
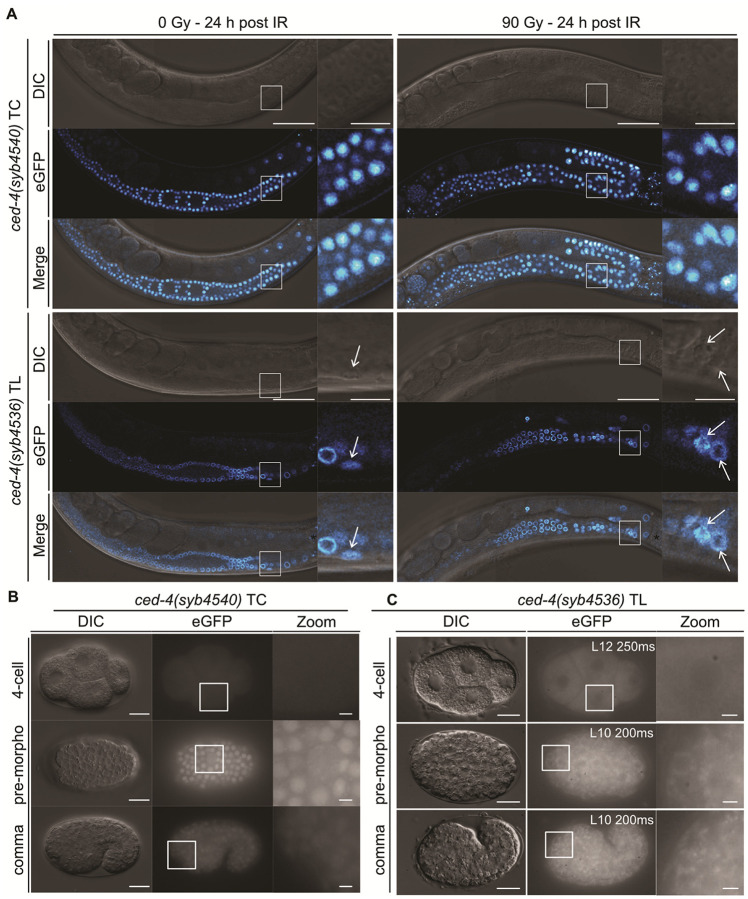
Expression pattern of the transcriptional *ced-4(syb4540)* and translational *ced-4(syb4536)* reporters in the germline and embryo. **(A)** Confocal live imaging of the transcriptional *ced-4(syb4540)* TC and translational *ced-4(syb4536)* TL reporters in the germline with and without IR. L4-staged worms were exposed to 0 Gy or 90 Gy and imaged 24 hours post-treatment. Insets show zoomed regions outlined by white boxes in the main panels. Asterisks (*) mark intestinal autofluorescence. White arrows indicate apoptotic germ-cell corpses. Scale bars: 50 μm (main panels), 10 μm (insets). Expression of the **(B)** transcriptional *ced-4(syb4540)* TC and **(C)**translational *ced-4(syb4536)* TL reporters during embryogenesis (4-cell, pre-morphogenesis, comma stage). Insets show zoomed regions outlined by white boxes in the main panels. Scale bars: 10 μm (main panels), 2 μm (insets). L indicates laser power, and ms indicates exposure time in ms.

To systematically study *ced-4* expression, we generated a transcriptional reporter, *ced-4(syb4540)*, replacing the entire coding sequence, and a translational reporter, *ced-4(syb4536)*, with a C-terminal linker::eGFP sequence to tag both CED-4 isoforms (Fig. S1C). The transcriptional *ced-4(syb4540)* reporter revealed ubiquitous *ced-4* expression in the germline (independently of IR) (Fig. 6A) and in embryos starting from the 8–12-cell stage (Fig. 6B). Analysis of the functional translational *ced-4(syb4536)* reporter (Fig. S8C+S10A) confirmed that CED-4 is ubiquitously expressed in the germline and localizes to the perinuclear membrane, consistent with previous observations (17) (Fig. 6A+S12A).

In early embryos (1–4-cell stage), CED-4 also localizes to the perinuclear membrane, whereas in later embryonic stages its localization became predominantly cytoplasmic (Fig. 6C) with high-resolution imaging showing colocalization of CED-4 with mitochondria (48) (Fig. S12B). The subcellular localization in early embryos likely persists from the germline, and the relocalization in later stage embryos (> 8–12-cell stage) may be necessary for proper apoptosis.

Altogether, these data demonstrate that CED-4 is ubiquitously expressed in the germline and embryo, and that its subcellular localization differs between these tissues. Our observation reconciles earlier findings (17, 38) by identifying a developmental switch in CED-4 localization from the perinuclear membrane to mitochondria.

### CED-9 is Ubiquitously Expressed and Forms Distinct Foci on Mitochondria in Both the Germline and the Embryo

**Figure 7:**
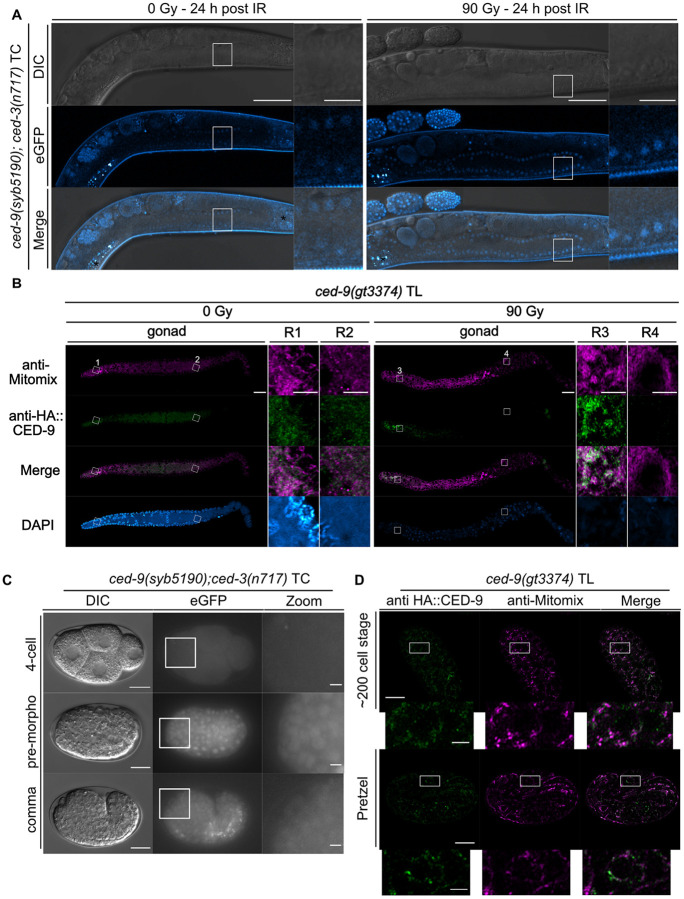
Expression pattern of the transcriptional *ced-9(syb5190)* TC and translational *ced-9(gt3374)* TL reporter in the germline and embryo. **(A)** Confocal live imaging of the transcriptional *ced-9(syb5190)* TC reporter in the germline with and without IR. L4-staged worms were exposed to 0 Gy or 90 Gy and imaged 24 hours post-treatment. Insets show zoomed regions outlined by white boxes in the main panels. Asterisks (*) mark intestinal autofluorescence. Scale bars: 50 μm (main panels), 10 μm (insets). **(B)** Airyscan imaging of immuno-stained germlines of the translational *ced-9(gt3374)* TL reporter with anti-HA, anti-Mitomix, and DAPI. L4-stage worms were exposed to 0 Gy or 90 Gy, and immuno-staining was performed 24 hours post-treatment. Insets show zoomed regions (R) outlined by white boxes in the main panels. Scale bars: 50 μm (main panels), 10 μm (insets). **(C)** Expression of the transcriptional *ced-9(syb5190)* TC reporter during embryogenesis (4-cell, pre-morphogenesis, comma stage). Insets show zoomed regions outlined by white boxes in the main panels. Scale bars: 10 μm (main panels), 2 μm (insets). **(D)** Airyscan imaging of immuno-stained embryos (200-cell stage and pretzel stage) of the translational *ced9(gt3374)*TL reporter with anti-HA and anti-Mitomix. Insets show zoomed regions outlined by white boxes in the main panels. Scale bars: 10 μm (main panels), 2 μm (insets). The transcriptional *ced9(syb5190)* TC reporter was generated in the *ced-3(n717)* background to ensure viability.

To examine *ced-9* expression, we generated a transcriptional reporter, *ced-9(syb5190)*, replacing the entire coding sequence. This reporter is engineered into the *ced-3(*lf*)* background to prevent apoptosis associated with *ced-9(syb5190)* (Fig. S1D). This reporter revealed ubiquitous *ced-9* expression in embryos and the germline (Fig. 7A + C). Attempts to generate a translational reporter by N-terminal eGFP tagging failed to produce viable homozygotes, indicating disruption of CED-9 function. As an alternative, we generated an N-terminal 3×HA-tagged reporter, *ced-9(gt3374)* (Fig. S1D). This reporter does not exhibit excessive embryonic apoptosis and embryonic lethality (Fig. S10A + C), indicating that it is functional in embryos. It is only partially functional in the germline, where it causes elevated apoptosis with or without IR (Fig. S8B) and a significantly reduced brood size (Fig. S10B). Immunostaining confirmed that in embryos, CED-9 localizes to distinct foci on mitochondria, consistent with previous reports (Fig. 7D) (17). Importantly, we further detected these foci throughout the entire germline in the absence of DNA damage (Fig. 7B+S14). Upon IR, CED-9 foci became spatially restricted to the mitotic and early transition zones and in a few late pachytene cells (Fig. 7B+S14). Together, these data show that *ced-9* is ubiquitously expressed in embryos and in the germline and that CED-9 protein forms distinct foci on mitochondria. In the germline, CED-9 localization becomes spatially restricted in response to DNA damage, revealing an additional layer of regulation.

## Discussion

**Figure 8:**
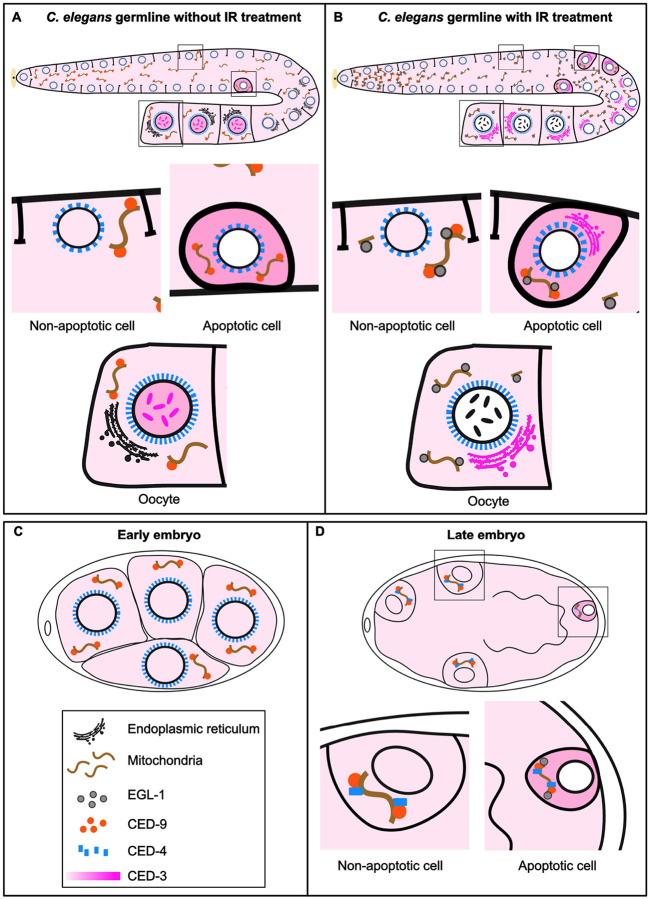
Model of the subcellular localization of apoptosis regulators in the germline, early and late embryos of *C. elegans*. **(A)** Non-irradiated germline. The core apoptosis regulators CED-9, CED-4, and CED-3 are ubiquitously expressed throughout the germline. CED-3 displays a diffuse localization in both the cytoplasm and the nucleus of germ cells, CED-4 exhibits a perinuclear distribution, and CED-9 forms foci on mitochondria. In oocytes, CED-3 becomes more concentrated around the condensed chromosomes. In cells undergoing physiological germ-cell apoptosis, CED-3 is retained in the cytoplasm but is no longer detectable in the nucleus**.(B)** Irradiated germline. Upon DNA damage, CED-4 and CED-9 maintain the same subcellular localization patterns as those observed under non-irradiated conditions. However, CED-9 appears less abundant in early and late pachytene. CED-3 redistributes to structures in the cytoplasm that are reminiscent of ER in a subset of germlines, in apoptotic cells and in early and late oocytes. EGL-1 is ubiquitously present throughout the germline but shows pronounced hyperaccumulation in a subset of apoptotic germ cell corpses. **(C)** Early embryo. In early embryos, CED-9, CED-4, and CED-3 display broadly ubiquitous localization patterns similar to those observed in the germline. At these stages, CED-4 remains predominantly associated with the perinuclear membrane. **(D)** Mid/Late embryo (≥8–12 cell stage). As embryogenesis progresses, CED-4 relocalizes to mitochondria. *egl-1* expression is restricted to cells fated to undergo apoptosis. In these cells, CED-3 is enriched in the cytoplasm and excluded from the nucleus.

In this study, we generated CRISPR/Cas9 endogenous transcriptional and translational reporters for all four apoptosis genes, *egl-1, ced-9, ced-4*, and *ced-3*, and systematically map their expression and subcellular localization in the *C. elegans* germline and embryos (Fig. 8). These analyses provide a framework for interpreting how distinct regulatory logics shape apoptotic outcomes in somatic lineages versus the germline.

### Distinct Modes of egl-1 Regulation Underly Somatic and Germline Apoptosis in C. elegans.

*C. elegans* developmental apoptosis follows a hardwired program. Our analysis demonstrates that *egl-1* transcription is restricted to lineages where cells are destined to die in wild-type animals. Previous work showed that *egl-1* mRNA is already present in the mother cell of cell destined to die (23) suggesting reporter detection may lag due to eGFP maturation kinetics or imaging sensitivity. Nevertheless, the spatial and temporal precision of *egl-1* activation supports its role as the decisive trigger for somatic apoptosis. In contrast, *ced-9*, *ced-4*, and *ced-3* are ubiquitously expressed in embryos, as previously reported (16, 26).

The germline follows a fundamentally different regulatory logic. Both transcriptional and translational *egl-1* reporters are strongly induced upon DNA damage. Consistent with previous work, this response is CEP-1/p53-dependent (30). Using targeted reporter designs, we identified intron1 of *egl-1* as a critical *cis*-regulatory element containing a CEP-1/p53-responsive regulatory module that enables widespread germline induction of *egl-1* following genotoxic stress. Some apoptotic germ cells exhibit pronounced EGL-1 hyperaccumulation. This accumulation may reflect either positive feedback between apoptotic and engulfment pathways, as previously suggested (62, 63), or delayed corpse clearance when high levels of DNA damage–induced apoptosis overwhelm the engulfment machinery. Surprisingly, *egl-1* induction is not restricted to late pachytene cells where apoptosis occurs, but extends across the entire germline. These results indicate that *egl-1* expression alone is insufficient to commit a germ cell to die, and imply that additional factors, potentially involving checkpoint signaling thresholds, mitochondrial physiological status, or the availability of downstream effectors, define which cells are competent to execute apoptosis. Supporting this model, *brc-1* and *syp-2* mutants show that distinct meiotic surveillance pathways converge on *egl-1* induction in both apoptotic and non-apoptotic cells. Thus, apoptosis in *C. elegans* is governed by two parallel but mechanistically distinct logics: (1) lineage-specific *egl-1* induction in embryos and (2) checkpoint-mediated activation of *egl-1* in the entire germline, where additional yet unidentified pathways restrict apoptotic execution. Several genes are required for DNA damage-induced germ-cell apoptosis without affecting DNA damage-induced *egl-1* transcription, and some act in a cell-nonautonomous manner. These include the IR-induced intestinal secreted SYSM-1 (64) and the scaffold protein KRI-1 (related to mammalian KRIT1/CCM1), which regulates MAP kinase signaling required for DNA damage–induced germ-cell apoptosis by controlling intestinal zinc sequestration (65). Finally, several DNA repair and DNA-damage response genes, whose loss blocks DNA damage-induced apoptosis without compromising *egl-1* induction, include the SIR-2 histone deacetylase (48), the GEN-1 Holliday junction resolvase (66), and Topoisomerase III (67). It will be interesting to test if any of these factors affect EGL-1 protein abundance, or post-translational modifications.

### Context-Dependent Localization of CED-9, CED-4, and CED-3 Shapes Apoptosis in C. elegans

Our CRISPR/Cas9 reporters uncovered context-dependent localization dynamics of CED-9, CED-4, and CED-3 across the germline and embryos. CED-9 localizes to mitochondria in embryos and in the germline as previously shown (16, 17). Interestingly, in the absence of DNA damage, CED-9 foci are observed throughout the germline. In contrast, upon IR, they become spatially restricted to the mitotic zone, early transition zone, and a few cells in the late pachytene zone. This apparent reduction in CED-9 abundance or distribution may contribute to apoptosis induction, as previously reported for physiological germ-cell apoptosis (68). Surprisingly, EGL-1 protein is detected throughout the germline upon DNA damage and exhibits mitochondrial localization, suggesting that even low levels of mitochondrial CED-9 are sufficient for EGL-1 recruitment, or that EGL-1 can be targeted to mitochondria independently of CED-9.

The localization of CED-4 has been debated for two decades. Antibody-based studies reported strong perinuclear enrichment in the germline (17), whereas earlier work from the Horvitz’s laboratory suggested mitochondrial localization in embryos (38). More recent studies using CRISPR/Cas9 reporter (16) and antibody staining (26) confirmed CED-4 mitochondrial localization during mid-embryogenesis but did not address CED-4 localization in early embryos. Our CRISPR/Cas9 reporters reconcile these findings by revealing a developmental transition: CED-4 localizes to the perinuclear membrane in the germline and in early embryos (1–4-cell stage) but progressively relocalizes to mitochondria as embryogenesis proceeds. This transition coincides with the onset of zygotic transcription and likely positions CED-4 for apoptosome assembly later during development. Consistent with Lambie and colleagues (16), we did not observe translocation of CED-4 to perinuclear membranes in embryonic apoptotic cells, as previously suggested (38).

The analysis of the CED-3 reporter revealed a sequence of regulated localization states. In late oocytes, CED-3 is enriched in the nucleus and occasionally near the chromatin, consistent with early immunostaining studies (22). Following DNA damage, CED-3 redistributes to ER-like cytoplasmic structures in a subset of germlines. This observation is particularly interesting given recent evidence that CED-3 protects worms against ER stress (69). During embryogenesis, apoptotic cells display striking ring-like cytoplasmic accumulations of CED-3, whereas adjacent surviving cells maintain a diffuse nuclear–cytoplasmic distribution. Altogether, these findings demonstrate that the apoptotic machinery, except for EGL-1, is broadly expressed during somatic development but functionally constrained by developmentally regulated subcellular dynamics. Rather than operating as a fixed linear pathway, apoptosis in *C. elegans* is governed by tissue-specific competence states, and stage-specific relocalization of key regulators, thereby refining the classical EGL-1–CED-9–CED-4–CED-3 model.

Importantly, these principles are likely conserved across metazoans. In the classical *C. elegans* somatic apoptosis model, CED-9 directly binds and sequesters CED-4 at the mitochondrial membrane, thereby preventing activation of the caspase CED-3. In contrast, in mammalian cells, BCL-2 family proteins do not directly bind APAF-1; instead, OMM permeabilization triggers cytochrome c release, promoting apoptosome assembly and caspase activation. Notably, in the *C. elegans* germline, the absence of stable CED-9-CED-4 colocalization brings the nematode apoptosis pathway closer to the mammalian paradigm, suggesting that apoptosome activation in the germline may rely on additional regulatory steps rather than simple sequestration by a BCL-2–like protein. In mammals, BCL-2 family proteins localize not only to mitochondria but also to the ER, where they regulate calcium signaling and ER stress independently of apoptosis (70, 71). Apaf-1 and caspases have similarly been implicated in non-apoptotic roles and are subject to spatial and contextual regulation (69, 72). Together, our findings in *C. elegans* support a conserved model in which apoptotic regulators are broadly expressed and multifunctional, with apoptosis emerging only when transcriptional activation, protein localization, and cellular competence converge. Rather than operating as a binary switch, the apoptotic machinery functions as a spatially and temporally regulated network, that ensures robustness against inappropriate cell loss while preserving rapid apoptotic capacity.

## Future Directions

A key unresolved question is why apoptosis occurs exclusively in late pachytene cells despite broad EGL1 induction upon IR; identifying the factors underlying this restricted competence remains an important direction for future studies. Integrating caspase activity sensors with high-resolution Airyscan and realtime spinning disk microscopy, along with single-cell transcriptomics and tissue-specific proteomics, will be critical for defining the molecular determinants of apoptosis competence in the germline.

## Conclusions

By generating endogenous reporters for the core apoptosis machinery, we provide a comprehensive view of apoptosis gene expression and localization in the *C. elegans* germline and embryos. Our findings reveal fundamental differences in apoptotic regulation between these tissues, and uncover changes in CED-3 and CED-4 localization that refine the classical model of apoptosis induction. Together, these insights reshape our understanding of apoptotic regulation and provide a foundation for further investigating the apoptotic and non-apoptotic functions of these conserved proteins.

## Supplementary Material

Supplementary Files

This is a list of supplementary files associated with this preprint. Click to download.

- GopakumaretalMovieS1.avi
- GopakumaretalMovieS2.avi
- GopakumaretalMovieS3.avi
- GopakumaretalMovieS4.avi
- GopakumaretalSupplementaryTables28.01.2026.pdf
- GopakumeretalSupplementaryFile.pdf

## Data Availability

All data and material used in this manuscript are available and can be requested from the corresponding authors.
